# Retinal arteriolar narrowing is associated with a 4-year risk of incident metabolic syndrome

**DOI:** 10.1038/nutd.2015.15

**Published:** 2015-06-22

**Authors:** K Saito, Y Kawasaki, Y Nagao, R Kawasaki

**Affiliations:** 1 Department of Ophthalmology, Shinoda General Hospital, Yamagata Japan; 2 Department of Public Health, Yamagata University Faculty of Medicine, Yamagata, Japan; 3 Department of Medical Check-up Center, Shinoda General Hospital, Yamagata, Japan

## Abstract

We aimed to determine whether retinal microvascular changes in vessel calibers at baseline are associated with the future risk of developing the metabolic syndrome over 4 years in an adult cohort of Japanese individuals (*n*=90) who attended a health-screening program. Retinal vessel caliber was calculated as the central retinal artery equivalent and vein equivalent (CRAE) from non-mydriatic digital fundus images using semiautomated standardized software. There were 18 cases (20%) that developed metabolic syndrome over 4 years. Narrower arteriolar caliber at baseline was associated with an increased risk of incident metabolic syndrome over 4 years after adjusting for potential confounding risk factors including individual cardiovascular risk factors related to the metabolic syndrome (adjusted odds ratio per 1 s.d. change in CRAE: 2.92, 95% confidence interval 1.03, 8.24; *P*=0.043). Persons with wider venular caliber at baseline were more likely to have incident metabolic syndrome, but this was not statistically significant. Retinal vascular caliber might provide independent and useful information to predict incident metabolic syndrome in a health screening program.

## Introduction

The metabolic syndrome, an accumulation of multiple cardiovascular risks, is now widely recognized as an important target of intervention to prevent cardiovascular disease both in clinical practice and in health screening program in Japan.^1^ There is also an emerging understanding that subtle retinal microvascular signs are associated with individual components of the metabolic syndrome. For example, retinal signs at baseline are associated with future development of hypertension^2^ or diabetes.^3^ Longitudinal association between baseline retinal vessel signs and incidence of obesity is still controversial; there was an association between wider retinal venular diameter and incidence of obesity in the Blue Mountains Eye study,^4^ although we could not confirm this in Japanese adults.^5^

Previously, we showed that retinal microvascular changes are associated with the presence of the metabolic syndrome.^6^ This finding was in concordance with other epidemiologic studies reporting that various retinal vascular signs are associated with the presence of the metabolic syndrome^7, 8^ or obesity.^5^ A drawback of these studies was that they are all cross-sectional studies; no temporal association between baseline retinal vascular changes and incidence of the metabolic syndrome has been investigated in a longitudinal study design to date.

In the present study, we aimed to determine whether retinal vessel caliber changes are associated with an increased risk of developing metabolic syndrome over 4 years in Japanese adults in the health screening program.

## Materials and methods

This study was approved by the Institutional review board of Shinoda General Hospital (Yamagata, Japan), and signed informed consent was obtained from each subject. In 2009, we recruited 90 healthy persons in an annual health screening program at Shinoda General Hospital (Yamagata, Japan). In 2013, we conducted a 4-year follow-up examination.

Diagnosis of metabolic syndrome was based on a joint interim statement of the International Diabetes Federation Task force on epidemiology and prevention,^9^ National Heart, Lung, and Blood Institute, American Heart Association, World Heart Federation, International Atherosclerosis Society, and International Association for the study of obesity. In brief, five risk factors of the metabolic syndrome were defined as follows:

- elevated triglyceride level: ⩾1.7 mmol l^−1^ (150 mg l^−1^) or the use of medication to treat elevated triglycerides;
- low high-density lipoprotein cholesterol (HDLc) levels: <1.0 mmol l^-1^ (40 mg dl^−1^) in men and 1.3 mmol l^−1^ (50 mg dl^−1^) in women, or the use of medication to treat low HDLc levels;
- elevated blood pressure: systolic blood pressure ⩾130 and/or diastolic blood pressure ⩾85 mm Hg^−1^ on examination, or the use of antihypertensive drugs to treat hypertension, or a history of hypertension;
- elevated fasting glucose: ⩾100 mg dl^−1^ on examination, or the use of medication to treat elevated blood glucose levels; and
- waist circumference: ⩾85 cm for men and ⩾90 cm for women.

A person was defined as having metabolic syndrome if 3 of 5 risk factors described above were present. In this study, the incidence of metabolic syndrome was defined if a subject was without metabolic syndrome at baseline but was having metabolic syndrome at the 4-year follow-up examination.

Retinal images were taken digitally without pharmacological pupil dilation with a TRC-NW200 camera (Canon Inc., Tokyo, Japan; and TRC, Topcon Inc., Tokyo, Japan). Retinal vascular diameters were measured using validated semiautomated computer-assisted imaging software (University of Wisconsin, Madison, WI, USA).^10^ The software automatically measured and calculated the caliber of arterioles and venules and summarized into the central retinal artery equivalent (CRAE) and central retinal vein equivalent (CRVE) using Knudtson's modification of the Parr–Hubbard formula,^11^ respectively.

### Statistical methods

To examine whether retinal vessel caliber is associated with the cumulative incidence of the metabolic syndrome, multiple logistic regression analysis was performed to estimate the odds ratios (ORs) independent of potential confounders per s.d. change in retinal vessel caliber. We determined the ORs adjusted for sex, age, and five individual components of metabolic syndrome at baseline examination; CRAE and CRVE were simultaneously included in the multivariate models, as recommended previously.^12^ On the basis of the multiple logistic models, we explored whether the addition of retinal vessel caliber into the model with risk factors of metabolic syndrome improves the diagnostic power by assessing the area under the curve (AUC) for receiver operating curve. All data were analyzed using Stata for Windows (version 13.1, StataCorp, College Station, TX, USA).

## Results

Characteristics of the study participants are described in Table 1. Over 4 years, there were 18 (20%) incidents of metabolic syndromes. After adjusting for age, gender, waist circumference, systolic blood pressure, triglycerides, HDL cholesterol, fasting glucose, and CRVE, persons with narrower CRAE at baseline were significantly more likely to have incident metabolic syndrome over 4 years (OR per 1 s.d. change: 2.92; 95% confidence interval (CI); 1.03–8.24). Persons with wider CRVE at baseline were more likely to have incident metabolic syndrome, but this was not statistically significant.

We further assessed the diagnostic power of using retinal vessel caliber information to identify persons at a higher risk of developing the metabolic syndrome. On the basis of the multiple logistic models, the AUC of the basic model adjusting for age, gender, and five components of metabolic syndrome was 0.833 (95% CI 0.738–0.928). When retinal vessel caliber measurements were added onto this model, AUC improved to 0.863 (95% CI 0.779–0.947), although this increase was not statistically significant (*P*=0.142). The AUC of the model with parameters from noninvasive examinations only (that is, without blood testing of glucose, triglycerides, and HDL cholesterol) without retinal vessel caliber was 0.800 (95% CI 0.705–0.895); the AUC improved to 0.813 (95% CI 0.719–0.906) when retinal vessel caliber was added on this. The AUC from the model with noninvasive examination with retinal vessel caliber was comparable to the basic model of age, gender, waist circumference, systolic blood pressure, triglycerides, HDL cholesterol, and fasting glucose (*P*=0.625) (Table 2).

## Discussion

In our cohort study of adult health check-up participants, we found the longitudinal association between retinal vessel caliber and incidence of the metabolic syndrome over 4 years. Narrowing of the retinal arterial diameter by 1 s.d. at baseline was associated with an almost threefold increased risk of 4-year incidence of metabolic syndrome, independent of age, gender, and five risk individual components of the metabolic syndrome. We can speculate that oxidative stress could be the underlying mechanism linking retinal arteriolar narrowing and incidence of the metabolic syndrome. It has been reported that higher glutathione peroxidase (GP × -3) activities, a biomarker of oxidative stress, was associated with narrower retinal arteriolar caliber in a cross-sectional analysis of 1224 individuals aged 60 years and over.^13^ Further studies are warranted to determine if the changes in the retinal vascular caliber is the result of oxidative stress or it is reflecting pathological mechanisms other than oxidative stress.

We also observed the highest AUC from the basic model including retinal vessel caliber measurement. Although the addition of retinal vessel caliber information was not significant, we also showed that the AUC from the model with noninvasive examination of age, gender, waist circumference, and systolic blood pressure plus retinal vessel calibers has an equivalent level of AUC compared with the fully adjusted model in identifying people at a high risk of developing the metabolic syndrome.

There are several limitations to our study: the sample size was small, the subjects were visitors to our medical check-up center and the study sample could be subject to selection bias. However, the strength of our study is its standardized assessment of metabolic syndrome and retinal vessel calibers. In addition, this is the first report of longitudinal association between retinal vessel caliber and the incident metabolic syndrome. To confirm the potential of this association, population-based studies with larger sample size and longer follow-up studies are needed.

In conclusion, we found the longitudinal association between retinal vessel caliber and the metabolic syndrome in this study. Retinal vascular assessment may provide additional information of vascular health and it can exist prior to developing the metabolic syndrome independent of the components of the metabolic syndrome.

## Figures and Tables

**Table 1:** Baseline characteristics of the study subjects

	*All participants (*n=*90)*	*Incident metabolic syndrome (*n=*18)*	*Not developing metabolic syndrome (*n=*72)*	P*-value*
Age at baseline, years old	48.9 (8.2)	48.9 (9.3)	48.0 (7.9)	0.673
Gender, female (%)	25 (27.8)	4 (22.2)	21 (29.2)	0.556
Systolic blood pressure, mmHg	122.7 (15.2)	129.4 (21.0)	121.0 (13.0)	0.035
Diastolic blood pressure, mmHg	76.7 (10.8)	78.5 (11.5)	76.3 (10.7)	0.436
Waist circumference, cm	80.4 (7.4)	86.3 (6.3)	79.0 (6.9)	<0.001
Triglycerides, mg dl^−1^	105.2 (51.9)	135.7 (51.4)	97.6 (49.4)	0.005
High-density lipoprotein cholesterol, mg dl^−1^	67.1 (16.2)	60.8 (13.7)	68.7 (16.5)	0.063
Fasting plasma glucose, mg dl^−1^	93.9 (8.6)	98.2 (9.8)	92.9 (8.1)	0.018
CRAE, μm	131.8 (14.4)	127.5 (10.1)	132.9 (15.1)	0.150
CRVE, μm	194.3 (18.0)	193.1 (14.9)	194.6 (18.8)	0.768

Abbreviations: CRAE, central retinal artery equivalent; CRVE, central retinal vein equivalent.

Data are shown as mean (s.d.).

**Table 2:** The AUC-ROC for the logistic regression models

	*AUC-ROC*	*(95% CI)*	P*-value*
			
*Basic model*			
Adjusted for age, gender, and five components of metabolic syndrome (waist circumference, systolic blood pressure, fasting plasma glucose, triglycerides, and high-density lipoprotein cholesterol)	0.833	(0.738, 0.928)	Reference
			
*Fully adjusted model*			
Adjusted for basic model plus retinal vessel calibers	0.863	(0.779, 0.947)	0.142
			
*Noninvasive examination model*			
Adjusted for noninvasive examination of derived parameters of age, gender, waist circumference, and systolic blood pressure plus retinal vessel calibers	0.813	(0.719, 0.906)	0.581

Abbreviations: AUC-ROC, area under the receiver operating curve; CI, confidence interval.
